# Long-term trends in smoking prevalence and its socioeconomic inequalities in Korea, 1992–2016

**DOI:** 10.1186/s12939-019-1051-x

**Published:** 2019-09-18

**Authors:** Youngs Chang, Hee-Yeon Kang, Dohee Lim, Hong-Jun Cho, Young-Ho Khang

**Affiliations:** 1 Department of Health Policy and Management, Seoul National University College of Medicine, 103 Daehak-ro, Jongno-gu, Seoul, 03080 South Korea; 2 Institute of Health Policy and Management, Seoul National University Medical Research Center, Seoul, South Korea; 3 Department of Family Medicine, Asan Medical Center, University of Ulsan College of Medicine, Seoul, South Korea

**Keywords:** Republic of Korea, Smoking, Socioeconomic factors

## Abstract

**Background:**

The aim of this study was to investigate long-term trends in smoking prevalence and its socioeconomic inequalities in Korea.

**Methods:**

Data were collected from 10 rounds of the Social Survey of Statistics Korea between 1992 and 2016. A total of 524,866 men and women aged 19 or over were analyzed. Age-adjusted smoking prevalence was calculated according to three major socioeconomic position indicators: education, occupational class, and income. The prevalence difference, prevalence ratio, slope index of inequality (SII), and relative index of inequality (RII) were calculated to examine the magnitude of inequality in smoking.

**Results:**

Smoking prevalence among men decreased from 71.7% in 1992 to 39.7% in 2016, while smoking prevalence among women decreased from 6.5% in 1992 to 3.3% in 2016. Socioeconomic inequalities in smoking prevalence according to the three socioeconomic position indicators were found in both men and women throughout the study period. In general, absolute and relative socioeconomic inequalities in smoking, measured by prevalence difference and prevalence ratio for education and occupational class, widened during the study period among Korean men and women. In men, the SII for income increased from 7.6% in 1999 to 10.8% in 2016 and the RII for income also increased from 1.11 in 1999 to 1.31 in 2016. In women, the SII for income increased from 0.1% in 1999 to 2.4% in 2016 and the RII for income increased from 1.39 in 1999 to 2.25 in 2016.

**Conclusion:**

Pro-rich socioeconomic inequalities in smoking prevalence were found in men and women. Socioeconomic inequalities in smoking have increased in parallel with the implementation of tobacco control policies. Tobacco control policies should be developed to decrease socioeconomic inequalities in cigarette use in Korea.

## Introduction

Cigarette smoking is a major risk factor for various chronic diseases, such as cancer (including, most notably, lung cancer), coronary heart disease, stroke, and chronic obstructive pulmonary disease [1–3]. According to the Global Burden of Disease Study, there were a total of 8.1 million deaths across the world from tobacco use in 2017, comprising 7.1 million deaths from smoking, 76,000 deaths from chewing tobacco, and 1.22 million deaths from exposure to secondhand smoke [4]. In response to this problem, the World Health Organization has been actively carrying out the Framework Convention on Tobacco Control with the goal of achieving a tobacco-free world [5]. Although smoking prevalence has sharply decreased in high-income countries such as the US and the United Kingdom over the past decades, the decrease in smoking prevalence in low- and middle-income countries remains unsatisfactory [6, 7]. Despite the decreasing trend in smoking prevalence across the world, the number of smokers is expected to reach 1.1 billion by the year 2025 [6].

Smoking prevalence is distributed disproportionately according to socioeconomic position (SEP) indicators such as education, occupation, and income, which in turn leads to inequalities in health outcomes. According to studies conducted in Western countries, a high smoking prevalence was observed among those with low SEP [8–13]. The mortality rate from smoking was also higher in low-SEP individuals [9]. According to the study by Kivimaki et al., elimination of cigarette use in all socioeconomic groups would reduce the absolute difference in deaths from coronary heart disease by 43% [12]. A study conducted in New Zealand observed an increase in socioeconomic inequalities in total mortality, in which the increase in inequalities in causes of death associated with smoking, such as lung cancer, was found to play a major role [13].

Studies have also been conducted in Korea to investigate socioeconomic inequalities in smoking prevalence. Various studies have found high smoking prevalence in both men and women with low SEP [14–21]. The contribution of smoking in explaining absolute inequalities in total mortality was reported to be greater than that of other risk factors such as hypertension, diabetes, and obesity [22]. Furthermore, despite the enforcement of tobacco control policies, such as increases in tobacco prices, smoke-free policies for indoor areas, and pictorial warning labels on tobacco products, there are reports that the relative inequalities in smoking prevalence have increased in Korea [14, 18].

Although studies have investigated trends in smoking prevalence and its socioeconomic inequalities in Korea [14, 15, 17, 18, 21], most of those studies were conducted within a period of about 10 years, and no studies have yet been conducted using long-term data extending for more than 20 years. Study results on long-term trends in smoking prevalence and its inequalities can have very important implications for establishing the directions of future tobacco control policies. This study analyzed long-term trends in smoking prevalence and its socioeconomic inequalities among men and women in Korea during the past 25 years from 1992 to 2016.

## Methods

This study was conducted based on multi-year cross-sectional surveys in Korea representing non-institutionalized adult men and women. Time trends of smoking prevalence and its socioeconomic inequalities were explored.

### Data

This study used data from the Social Survey of Statistics Korea, which are representative of national statistics. The Social Survey has been conducted annually since 1977 by Statistics Korea, the governmental agency for official statistics in Korea [14, 23]. The Social Survey contains 10 sections, including family, income and consumption, labor, education, health, environment, welfare, culture and leisure, safety, and social participation. The section on health is included on the survey once every 2–4 years. The sample was selected by a stratified probability sampling method. Since 2009, the annual target sample size has been about 37,000 members of 17,000 to 18,000 households that are sampled nationwide, which corresponds to half of the sample size of the years until 2008. The survey is carried out at the home of interviewees with face-to-face interviews by well-trained interviewers from Statistics Korea [23]. The response rates for the Social Survey have been about 70–80%. The Social Survey of Statistics Korea was the sole source of publicly available data suitable for investigating trends in smoking prevalence and its socioeconomic inequalities in Korea for a period of more than 20 years. Data were collected from 10 rounds of the Social Survey that included questions on health behaviors, which were conducted in 1992, 1995, 1999, 2003, 2006, 2008, 2010, 2012, 2014, and 2016. Data before 1992 were not available at the time of this study. Study subjects were designated as male and female adults aged 19 years and above. The age of 19 is the minimum legal age for purchasing and smoking tobacco products in Korea. The total sample size for this study was 524,866 subjects, excluding subjects with any missing data regarding socioeconomic position and smoking status. A total of 3.2% of the original samples were excluded due to missing data for these variables.

### Outcome variable

Current smoking status was used as the outcome variable. Because the Social Survey did not include a question on whether an individual had smoked over 5 packs (100 cigarettes) in his or her lifetime, individuals who responded “Yes” to the question “Do you currently smoke?” were defined as current smokers. The data needed to calculate pack-years were not available in the Social Survey.

### Socioeconomic position (SEP) indicators

Education, occupation, and income, the most frequently used indicators in the literature, were used as SEP indicators in this study. The question “What is the highest level of education you have attained?” was used to determine educational attainment. Education level was divided into graduation from high school or less and graduation from college or higher. The question “What is your current occupation?” was used to determine occupation. The interviewees were asked to indicate their current position and type of occupation. The well-trained interviewers classified the type of occupation based on the Korean Standard Classification of Occupations. Occupational class was divided into manual, non-manual, and others. Manual occupations comprised service workers; sales workers; skilled agricultural, forestry, and fishery workers; craft and related trades workers; equipment, machine operating, and assembling workers; and elementary workers. Non-manual occupations comprised managers, professionals and related workers, and clerks, and other occupations comprised the armed forces and groups with no income (housewives and students). In the Social Survey, respondents were asked to provide their total household income. Equivalized income, calculated by dividing the self-reported total household income by the square root of household size, was used to measure income. The equivalized income was then divided into three levels at the nearest tertile points to allow stable outcome calculations in consideration of the sample size for each year. Because information on income was only available in the data from 1999, 2006, 2008, 2010, 2012, 2014, and 2016, analyses of smoking prevalence and its socioeconomic inequalities according to income were only conducted for the corresponding years.

### Statistical analysis

Male and female adults aged 19 years and above were analyzed, with separate analyses conducted for male and female subjects. Age-standardized smoking prevalence was calculated for each year, and the 2005 population census was used for the standard population. According to Khang et al., changing trends in smoking prevalence and its inequalities in Korea varied by age [17]; therefore, the age-standardized smoking prevalence was calculated for age groups of 19–34 years, 35–49 years, 50–64 years, and 65 years and above. Age-standardized smoking prevalence according to socioeconomic position was also calculated for each year. The slope index of inequality (SII) (for income) and prevalence difference (PD) (for education and occupation) were calculated to determine absolute magnitudes of smoking inequalities, using a binomial model with the identity link [24]. Meanwhile, the relative index of inequality (RII) (for income) and prevalence ratio (PR) (for education and occupation) were computed to measure relative inequalities in log-binomial regression analyses [24]. Using PROC GENMOD of SAS version 9.4 (SAS Institute, Inc., Cary, NC, USA), the LINK IDENTITY option was used for PD and SII, while the LINK LOG option was employed for PR and RII. All analyses were conducted using weighted samples. The SII and RII were obtained by calculating the relative position using the cumulative distribution of the midpoint value of each age-adjusted group [24, 25]. Theoretically, SII is interpreted as the absolute difference in health status between the lowest-ranking and the highest-ranking groups [25, 26]. The RII is the ratio of prevalence between the highest-ranking group and the lowest-ranking group. The time trends of PR, PD, RII and SII were estimated by examining the *p*-values for an interaction term of the SEP indicators and the year variables in the statistical models. Since two educational categories were employed in this study, we only estimated PD and PR for education, rather than RII and SII.

## Results

The general characteristics of the study subjects are presented in Additional file 1: Table S1. There was a total of 246,096 men (46.9%) and 278,770 women (53.1%). The percentage of subjects aged 65 years and above increased each year. Among the total subjects, the percentage of subjects aged 65 years and above increased from 6.8% (1992) to 14.4% (2016) in men, and from 10.6% (1992) to 19.1% (2016) in women. There was a prominent increase in the percentage of subjects with an education level of college or higher, especially in women. The percentage of women with an education level of college or higher increased by approximately 3.1 times, from 13.8% in 1992 to 42.6% in 2016. The percentage of subjects with a non-manual occupation also increased among the total subjects; in men, the percentage increased from 18.1% in 1992 to 27.3% in 2016, and in women, from 10.4% in 1992 to 21.1% in 2016. Among the total study subjects, the number of smokers was significantly higher in men than in women. The percentage of smokers among the total subjects showed a decreasing trend over the years in both men and women.

**Fig. 1:**
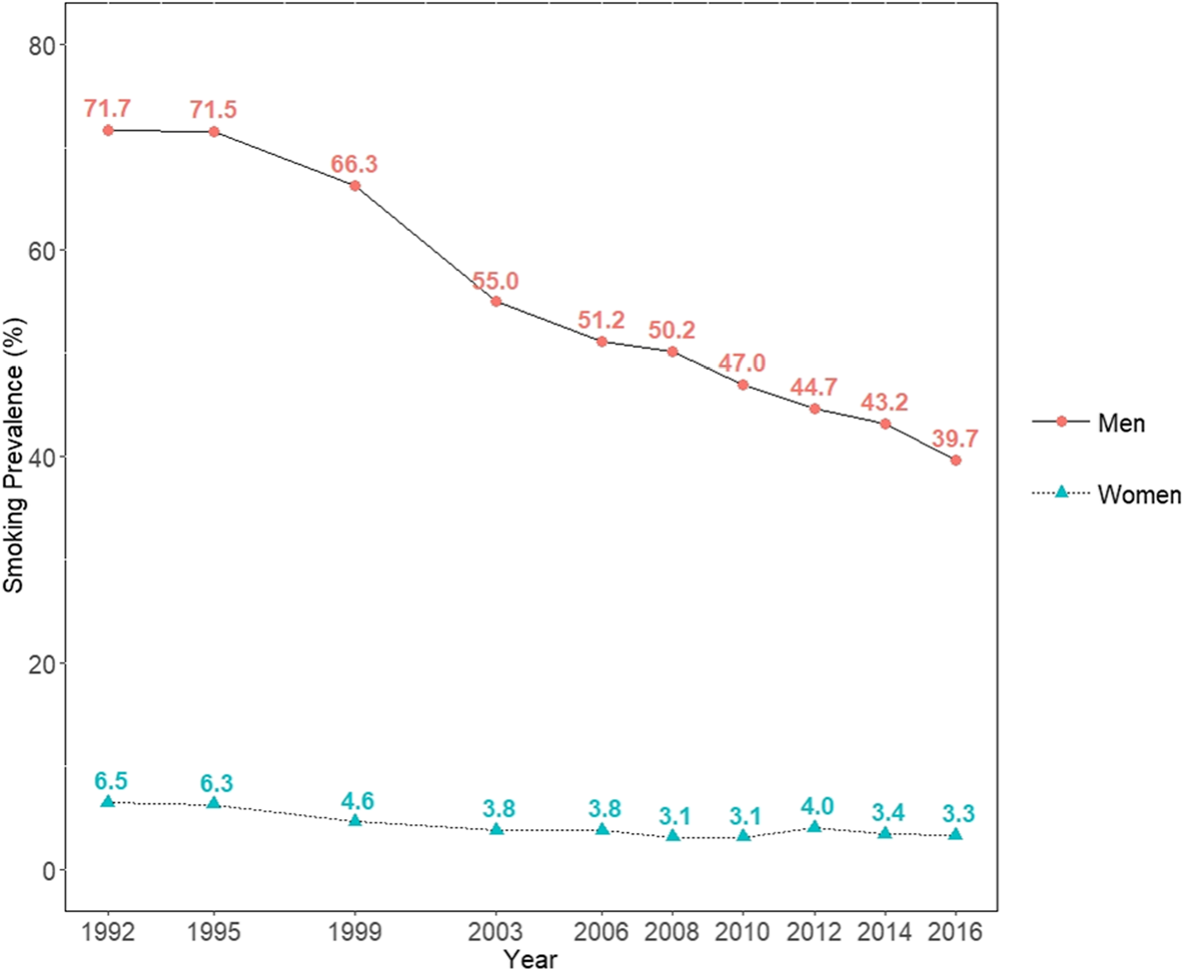
Trends in smoking prevalence between 1992 and 2016 among Korean men and women: Results from the Social Survey of Statistics Korea

Figure 1 and Additional file 1: Table S2 present the trend in age-standardized smoking prevalence over time. Smoking prevalence in men and women decreased from 71.7% (95% CI, 71.1 to 72.2%) in 1992 to 39.7% (95% CI, 38.7 to 40.6%) in 2016, and from 6.5% (95% CI, 6.3 to 6.8%) in 1992 to 3.3% (95% CI, 2.9 to 3.6%) in 2016, respectively. There was an especially prominent decrease between 1999 and 2003 in men, of more than 10% during 4 years. Although smoking prevalence in women decreased during the 10-year period starting in 1992, it remained virtually unchanged for the next 10 years after 2003.

**Fig. 2:**
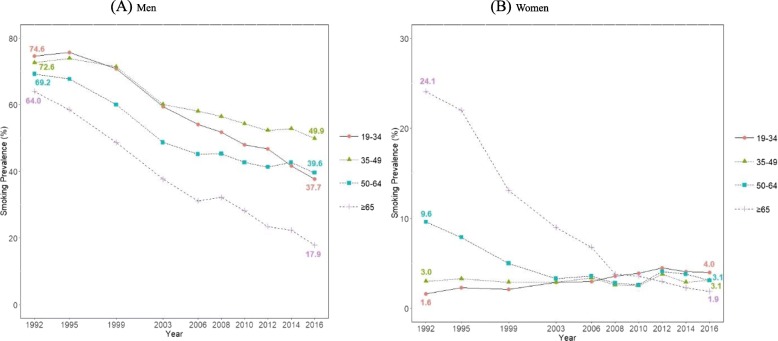
Trends in age group-specific smoking prevalence between 1992 and 2016 among Korean men (**a**) and women (**b**): Results from the Social Survey of Statistics Korea

Figure 2 presents the age group-specific smoking prevalence, with age groups of 19–34 years, 35–49 years, 50–64 years, and 65 years and above. During the past 25 years, smoking prevalence in men decreased in all age groups. Although smoking prevalence in men continued to decrease during the most recent 10 years in the age groups of 19–34 years and 65 years and above, it either remained stationary or showed a minimal reduction in the age groups of 35–49 years and 50–64 years. Although smoking prevalence in women aged 65 years and above showed a huge decrease from 24.1% (95% CI, 22.7 to 25.4%) to 1.9% (95% CI, 1.4 to 2.5%), smoking prevalence in women aged 19–34 years increased from 1.6% (95% CI, 1.4 to 1.9%) to 4.0% (95% CI, 3.2 to 4.8%). Smoking prevalence in women aged 35–49 years remained in the range of 3%, without significant changes in the past 25 years. Despite the decrease in smoking prevalence between 1992 and 2003 in women aged 50–64 years, there was no additional decrease in the following years.

**Fig. 3:**
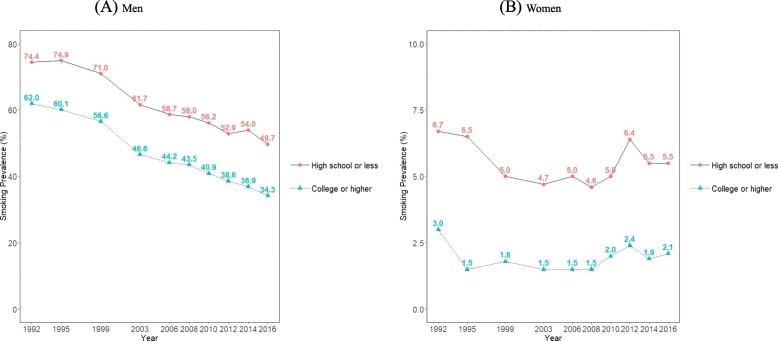
Trends in age-standardized smoking prevalence according to educational attainment between 1992 and 2016 among Korean men (**a**) and women (**b**): Results from the Social Survey of Statistics Korea

Figure 3 and Additional file 1: Table S2 present the age-standardized smoking prevalence calculated according to education level. In both men and women, smoking prevalence in participants who graduated from high school or less was higher than in subjects who graduated from college or higher, and this trend was consistent from 1992 to 2016. Between 1992 and 2016, smoking prevalence in men who graduated from high school or less and from college or higher decreased from 74.4% (95% CI, 73.8 to 75.0%) to 49.7% (95% CI, 47.9 to 51.4%), and from 62.0% (95% CI, 60.3 to 63.8%) to 34.3% (95% CI, 33.1 to 35.6%), respectively. Accordingly, the absolute difference in smoking prevalence between the two education groups increased from 12.4%p in 1992 to 15.4%p in 2016. Table 1 presents the PD and PR for education in men. The PD for education in men increased from 11.3%p (95% CI, 10.1 to 12.4%p) in 1992 to 12.9%p (95% CI, 11.4 to 14.3%p) in 2016 (p for trend = 0.0030). The PR for education in men also increased from 1.17 (95% CI, 1.15 to 1.19) in 1992 to 1.43 (95% CI, 1.38 to 1.49) (p for trend < 0.0001).

**Table 1:** Trends in the prevalence difference (PD), prevalence ratio (PR), slope index of inequality (SII), and relative index of inequality (RII) of smoking and its 95% confidence intervals (CI) according to education, occupational class, and income tertile: Results from the Social Survey of Statistics Korea

	1992	1995	1999	2003	2006	2008	2010	2012	2014	2016	P for trend
Men											
Education											
PD	11.3 (10.1–12.4)	12.5 (11.4–13.6)	13.2 (12.0–14.4)	15.2 (14.0–16.4)	14.6 (13.4–15.8)	14.3 (12.8–15.8)	14.0 (12.4–15.6)	13.8 (12.2–15.4)	15.5 (13.9–17.1)	12.9 (11.4–14.3)	0.003
PR	1.17 (1.15–1.19)	1.19 (1.17–1.21)	1.21 (1.19–1.23)	1.31 (1.28–1.34)	1.33 (1.29–1.36)	1.32 (1.28–1.36)	1.35 (1.30–1.40)	1.36 (1.31–1.41)	1.46 (1.40–1.51)	1.43 (1.38–1.49)	< 0.0001
Occupation											
PD	10.7 (9.5–11.9)	10.5 (9.2–11.8)	10.6 (9.3–11.9)	11.8 (10.4–13.1)	13.2 (11.8–14.5)	12.5 (10.8–14.1)	12.9 (11.0–14.7)	14.1 (12.3–16.0)	15.5 (13.7–17.3)	11.9 (10.1–13.6)	0.0034
PR	1.16 (1.14–1.18)	1.15 (1.13–1.18)	1.16 (1.09–1.02)	1.22 (1.20–1.25)	1.28 (1.25–1.32)	1.26 (1.23–1.31)	1.3 (1.25–1.35)	1.35 (1.30–1.41)	1.43 (1.37–1.49)	1.36 (1.30–1.42)	< 0.0001
Income											
SII			7.6 (5.6–9.5)		13.5 (11.4–15.6)	11.1 (8.5–13.7)	14.5 (11.7–17.3)	12.9 (10.1–15.7)	12.0 (9.3–14.8)	10.8 (8.1–13.5)	< 0.0001
RII			1.11 (1.08–1.15)		1.28 (1.23–1.34)	1.23 (1.17–1.29)	1.34 (1.27–1.43)	1.32 (1.24–1.40)	1.31 (1.23–1.40)	1.31 (1.22–1.41)	< 0.0001
Women											
Education											
PD	1.3 (0.9–1.6)	1.6 (1.2–2.0)	1.9 (1.6–2.3)	2.4 (2.0–2.7)	2.7 (2.3–3.2)	2.4 (1.9–2.9)	2.2 (1.7–2.7)	3.0 (2.4–3.6)	3.4 (2.9–4.0)	2.3 (1.9–2.8)	< 0.0001
PR	2.18 (1.68–2.82)	2.18 (1.76–2.69)	3.01 (2.32–3.92)	3.13 (2.59–3.79)	2.86 (2.40–3.42)	2.87 (2.32–3.55)	2.86 (2.29–3.58)	2.86 (2.36–3.48)	2.96 (2.41–3.64)	2.89 (2.37–3.53)	< 0.0001
Occupation											
PD	2.1 (1.7–2.6)	2.4 (1.8–2.9)	2.4 (1.9–2.9)	2.4 (1.9–2.8)	2.0 (1.4–2.5)	3.3 (2.6–4.1)	2.1 (1.3–2.8)	3.5 (2.7–4.4)	3.3 (2.5–4.1)	3.0 (2.3–3.8)	< 0.0001
PR	1.54 (1.20–1.98)	2.63 (1.84–3.77)	2.8 (2.06–3.82)	2.81 (2.22–3.56)	1.84 (1.52–2.24)	3.01 (2.33–3.88)	2.10 (1.60–2.75)	2.62 (2.06–3.34)	3.04 (2.33–3.98)	2.62 (2.05–3.34)	< 0.0001
Income											
SII			0.1 (0.0–0.8)		3.2 (2.5–3.9)	2.8 (1.9–3.6)	2.4 (1.5–3.3)	4.5 (3.5–5.6)	3.2 (2.3–4.2)	2.4 (1.5–3.3)	0.0003
RII			1.39 (1.16–1.66)		2.46 (2.01–3.00)	2.45 (1.85–3.23)	2.21 (1.63–2.98)	3.11 (2.36–4.11)	2.73 (2.04–3.66)	2.25 (1.68–3.03)	0.0008

Notes: The reference in estimating prevalence difference and prevalence ratio was college or higher for education and non-manual for occupational class

As shown in Fig. 3 and Additional file 1: Table S2, between 1992 and 2016, smoking prevalence in women who graduated from high school or less and from college or higher decreased from 6.7% (95% CI, 6.4 to 6.8%) to 5.5% (95% CI, 4.5 to 6.5%), and from 3.0% (95% CI, 1.3 to 4.6%) to 2.1% (95% CI, 1.4 to 2.8%), respectively; there were no significant changes in the absolute difference, which decreased from 3.7%p in 1992 to 3.4%p in 2016. However, results on PD and PR showed significantly increasing trends of absolute and relative inequalities in smoking by education among women (Table 1). The PD for education in women increased from 1.3%p (95% CI, 0.9 to 1.6%p) in 1992 to 2.3%p (95% CI, 1.9 to 2.8%p) in 2016 (p for trend < 0.0001). The PR for education in women also increased from 2.18 (95% CI, 1.68 to 2.82) in 1992 to 2.89 (95% CI, 2.37 to 3.53) in 2016 (p for trend < 0.0001).

**Fig. 4:**
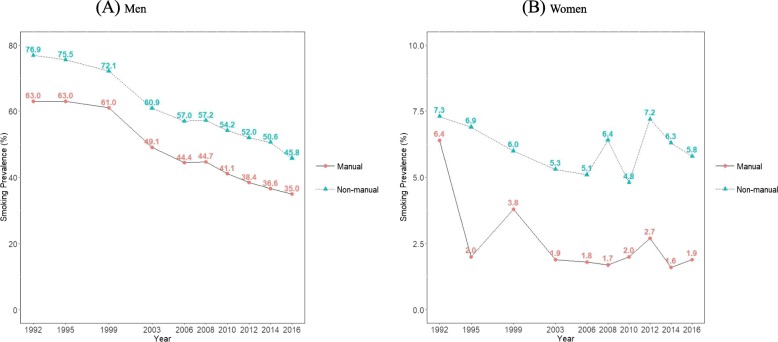
Trends in age-standardized smoking prevalence according to occupational class between 1992 and 2016 among Korean men (**a**) and women (**b**): Results from the Social Survey of Statistics Korea

Figure 4 and Additional file 1: Table S2 present the age-standardized smoking prevalence calculated according to occupation. In both men and women, smoking prevalence in participants with non-manual occupations was lower than in those with manual occupations. Between 1992 and 2016, smoking prevalence in men with non-manual occupations and manual occupations decreased from 63.0% (95% CI, 61.1 to 65.0%) to 35.0% (95% CI, 32.9 to 37.2) and from 76.9% (95% CI, 76.2 to 77.6%) to 45.8% (95% CI, 44.3 to 47.2%), respectively. Table 1 presents the PD and PR for occupational class (manual versus non-manual) in men. The PD for occupational class in men increased from 10.7%p (95% CI, 9.5 to 11.9%p) in 1992 to 11.9%p (95% CI, 10.1 to 13.6%p) in 2016 (p for trend = 0.0034). The PR for occupational class in men also increased from 1.16 (95% CI, 1.14 to 1.18) in 1992 to 1.36 (95% CI, 1.30 to 1.42) in 2016 (p for trend < 0.0001).

As shown in Fig. 4 and Additional file 1: Table S2, between 1992 and 2016, smoking prevalence in women with non-manual occupations and manual occupations decreased from 6.4% (95% CI, 4.3 to 8.5%) to 1.9% (95% CI, 1.3 to 2.4%) and from 7.3% (95% CI, 6.8 to 7.8%) to 5.8% (95% CI, 4.7 to 6.9%), respectively; the absolute difference in smoking prevalence increased from 0.9%p in 1992 to 3.9%p in 2016. Table 1 also showed increasing inequalities in smoking by occupational class among Korean women. The PD for occupational class in women increased from 2.1%p (95% CI, 1.7 to 2.6%p) in 1992 to 3.0%p (95% CI, 2.3 to 3.8%p) in 2016 (p for trend < 0.0001). The PR for occupational class in women increased from 1.54 (95% CI, 1.20 to 1.98) in 1992 to 2.62 (95% CI, 2.05 to 3.34) in 2016 (p for trend < 0.0001).

**Fig. 5:**
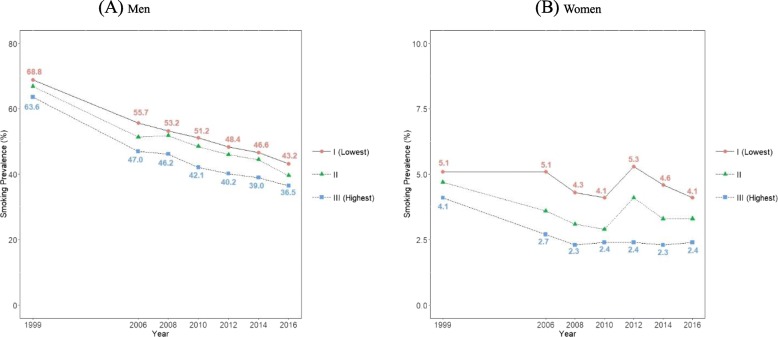
Trends in age-standardized smoking prevalence according to income tertile between 1999 and 2016 among Korean men (**a**) and women (**b**): Results from the Social Survey of Statistics Korea

Figure 5 and Additional file 1: Table S2 present age-standardized smoking prevalence calculated according to income. Inequalities were found in smoking prevalence among income levels in both men and women throughout the study period. Between 1999 and 2016, smoking prevalence of the lowest and highest income levels in men decreased from 68.8% (95% CI, 67.7 to 69.8%) to 43.2% (95% CI, 41.6 to 44.9%), and from 63.6% (95% CI, 62.5 to 64.6%) to 36.5% (95% CI, 34.8 to 38.1%), respectively, and the difference tended to increase from 5.2%p in 1999 to 6.7%p in 2016. Among women, between 1999 and 2016, smoking prevalence of the lowest and highest income levels decreased from 5.1% (95% CI, 4.7 to 5.5%) to 4.1% (95% CI, 3.4 to 4.8%), and from 4.1% (95% CI, 3.7 to 4.5%) to 2.4% (95% CI, 1.9 to 2.9%), respectively, and the difference increased from 1.0%p in 1999 to 1.7%p in 2016. Table 1 presents the SII and RII according to income level in each year. Between 1999 and 2016, the SII and RII increased in both men and women. In men, the SII increased from 7.6%p (95% CI, 5.6 to 9.5%p) in 1999 to 10.8%p (95% CI, 8.1 to 13.5%p) in 2016 (p for trend < 0.0001). In men, the RII increased from 1.11 (95% CI, 1.08 to 1.15) in 1999 to 1.31 (95% CI, 1.22 to 1.41) in 2016 (p for trend< 0.0001). In women, the SII increased from 0.1%p (95% CI, 0 to 0.8%p) in 1999 to 2.4%p (95% CI, 1.5 to 3.3%p) in 2016 (p for trend = 0.0003), and the RII increased from 1.39 (95% CI, 1.16 to 1.66) in 1999 to 2.25 (95% CI, 1.68 to 3.03) in 2016 (p for trend = 0.0008).

## Discussion

This study investigated long-term trends in smoking prevalence and its socioeconomic inequalities in Korea using data from the Social Survey of Statistics Korea. Despite the decreases in smoking prevalence in Korea between 1992 and 2016, socioeconomic inequalities in smoking were evident in both men and women over the study period. Both absolute and relative magnitudes of socioeconomic inequalities in smoking have increased in parallel with the implementation of tobacco control policies.

The results of this study showed that smoking prevalence in both men and women decreased. Following the enactment of the National Health Promotion Act in 1995, various tobacco control policies, such as smoke-free policies for large buildings, bans on tobacco advertising, anti-smoking media campaigns, smoke-free policies for all public places including restaurants, pictorial warning labels on tobacco products, and increases in tobacco prices have been actively pursued in Korea [14, 18, 27]. Smoking prevalence decreased in both men and women, particularly between 1999 and 2003, with a prominent change in the elderly groups of both sexes. This result suggests that the anti-smoking media campaigns featured through TV Star starting in 2000 were especially effective in elderly smokers [17, 28]. In recent years, the substantial increase in tobacco prices enforced in 2015 seems to have contributed to the decrease in smoking prevalence between 2014 and 2016.

Smoking prevalence decreased in men in all age groups; however, the decrease was especially prominent in the age group of 19–34 years and in the age group of 65 years and above. The decrease in smoking prevalence in the age group of 19–34 years reflects a decline in the smoking initiation rate, and therefore might demonstrate the effect of anti-smoking policies targeted towards teenagers in Korea [29]. Meanwhile, the decrease in smoking prevalence in the elderly age group can be interpreted as indicating that a greater number of elderly individuals discontinued smoking due to health concerns than was the case in the past, in accordance with changes in social norms, such as increased awareness via media campaigns [17].

The results of the analysis presented herein showed that the age gradients in smoking prevalence among women were reversed over time. Specifically, while smoking prevalence in the elderly age group was higher than in the younger age group in 1992, this phenomenon was reversed in 2016. This phenomenon of reversal in the smoking prevalence according to age among women has also been observed in Japan [30]. Among women, smoking is closely associated with gender roles and social norms. A high smoking prevalence in elderly women has been commonly observed in Asian countries [17, 31–35], which is contrary to the phenomenon observed in Western countries, where high smoking prevalence in women is observed in the younger age group [36]. Traditionally, the social stigma of smoking was powerful, especially in young women of childbearing age, and the social pressure upon them to quit smoking was predominant. However, elderly women were more liberated from such social pressures, especially patriarchal restrictions on women smoking, for many of them were either widowed or divorced [31]. Nonetheless, it appears that the trends in smoking prevalence among age groups observed in Western countries are appearing in Korea as well, in accordance with changes in the perceptions of women’s gender roles and changes in social norms regarding smoking in young women [17, 31].

Inequalities in smoking prevalence were present according to education, occupation, and income, and were continuously observed for the past 25 years in both Korean men and women. In particular, an increasing trend in the absolute magnitude of socioeconomic inequalities in smoking prevalence between socioeconomic groups was observed. Our analyses of PD and PR showed increasing time trends in both absolute and relative inequalities in smoking according to education and occupational class (Table 1). Although previous studies conducted in Korea have regularly reported an increasing trend in socioeconomic inequalities in smoking [15, 17, 18], this is the first study to report long-term changes in inequalities in smoking throughout a period of 25 years. This study also presents recent changes in the magnitude of inequalities in smoking; for example, the SII of inequalities in smoking according to income level decreased in both men and women between 2014 and 2016, and the RII also decreased in women (Table 1). It may be possible to attribute this trend to anti-smoking policies (increase in tobacco price from KRW 2500 (USD 2.3) to KRW 4500 (USD 4.0) per pack, enforcement of smoke-free policies for indoor areas) implemented in 2015 [14].

A strength of this study is that it is the first to report long-term trends of socioeconomic inequalities in smoking for more than 20 years in Korea. The Social Survey was the only available data source for the analysis of long-term trends in smoking prevalence and its socioeconomic inequalities in Korea. Additionally, we presented inequalities in smoking according to major three SEP indicators: education, occupation, and income. This study also has certain limitations. The variables used in this study were based on self-reporting. The self-reported smoking prevalence among Korean women may have been underestimated [37]. Furthermore, the information on household income may not have been optimal. However, prior Korean studies showed that self-reported income was a good predictor of health outcomes such as mortality [38]. In this study, we only presented time trends of inequalities in smoking. A decomposition analysis may provide additional information regarding the determinants of inequalities in smoking. In addition, analyses of other aspects of smoking behaviors such as smoking initiation, quitting, and hardcore smoking may provide detailed information on trends in smoking behaviors and their inequalities.

In conclusion, the smoking prevalence has substantially decreased between 1992 and 2016, especially among elderly Korean men. However, young Korean women recorded increases in smoking prevalence. Tobacco control policies to address these long-term trends of subgroup-specific smoking prevalence should be developed in the future. Although the smoking prevalence in Korea decreased between 1992 and 2016, inequalities in smoking were evident over the period. Both absolute and relative inequalities, measured by PD, SII, PR, and RII, increased despite the enforcement of various tobacco control policies since 1992, and these results therefore indicate that those policies were not effective in reducing inequalities in smoking prevalence. Our study results demonstrate the necessity of tobacco control policies designed to reduce inequalities in smoking, including additional increases in tobacco prices. Additionally, an investigation of long-term trends in smoking prevalence and its inequalities will provide a fundamental basis for determining the direction of new tobacco control policies to be implemented in the future.

## Supplementary information

**Additional file 1: Table S1.** Numbers and proportions (standard error) of subjects and smokers according to sex, age group, education, occupation, and income tertile: Results from the Social Survey of Statistics Korea. **Table S2.** Trends in age-standardized smoking prevalence (%) and its 95% confidence intervals (CI) according to sex, age group, education, occupation, and income tertile: Results from the Social Survey of Statistics Korea. (DOCX 51 kb)

## Data Availability

The datasets used and/or analyzed during the current study are publicly available from Statistics Korea (https://mdis.kostat.go.kr).

## Abbreviations

CI: confidence intervals
PD: prevalence difference
PR: prevalence ratio
RII: relative index of inequality
SEP: socioeconomic position
SII: slope index of inequality

## References

[1] Department of Health and Human Services (2014). The health consequences of smoking—50 years of Progress: a report of the surgeon General. Atlanta, GA: US Department of Health and Human Services, Centers for Disease Control and Prevention.

[2] Hackshaw A, Morris JK, Boniface S, Tang JL, Milenkovic D (2018). Low cigarette consumption and risk of coronary heart disease and stroke: meta-analysis of 141 cohort studies in 55 study reports. BMJ.

[3] GBD Tobacco Collaborators (2017). Smoking prevalence and attributable disease burden in 195 countries and territories, 1990–2015: a systematic analysis from the Global Burden of Disease Study 2015. Lancet.

[4] GBD 2017 Risk Factor Collaborators (2018). Global regional, and national comparative risk assessment of 84 behavioural, environmental and occupational, and metabolic risks or clusters of risks for 195 countries and territories, 1990–2017: a systematic analysis for the Global Burden of Disease Study 2017. Lancet.

[5] World Health Organization. WHO Framework Convention on Tobacco Control. http://www.who.int/fctc/text_download/en/index.html. Accessed December 3, 2018.

[6] Bilano V, Gilmour S, Moffiet T, d'Espaignet ET, Stevens GA, Commar A (2015). Global trends and projections for tobacco use, 1990–2025: an analysis of smoking indicators from the WHO Comprehensive information Systems for Tobacco Control. Lancet.

[7] Ng M, Freeman MK, Fleming TD, Robinson M, Dwyer-Lindgren L, Thomson B (2014). Smoking prevalence and cigarette consumption in 187 countries, 1980–2012. JAMA.

[8] Bosdriesz JR, Willemsen MC, Stronks K, Kunst AE (2015). Socioeconomic inequalities in smoking cessation in 11 European countries from 1987 to 2012. J Epidemiol Community Health.

[9] Gregoraci G, van Lenthe FJ, Artnik B, Bopp M, Deboosere P, Kovács K, et al. Contribution of smoking to socioeconomic inequalities in mortality: a study of 14 European countries, 1990–2004. Tob Control. 2016. 10.1136/tobaccocontrol-2015-052766 27122064

[10] Hu Y, van Lenthe FJ, Platt S, Bosdriesz JR, Lahelma E, Menvielle G (2017). The impact of tobacco control policies on smoking among socioeconomic groups in nine European countries, 1990–2007. Nicotine Tob Res.

[11] Mackenbach JP, Stirbu I, Roskam A-JR, Schaap MM, Menvielle G, Leinsalu M (2008). Socioeconomic inequalities in health in 22 European countries. N Engl J Med.

[12] Kivimäki M, Shipley MJ, Ferrie JE, Singh-Manoux A, Batty GD, Chandola T (2008). Best-practice interventions to reduce socioeconomic inequalities of coronary heart disease mortality in UK: a prospective occupational cohort study. Lancet.

[13] Fawcett J, Blakely T (2007). Cancer is overtaking cardiovascular disease as the main driver of socioeconomic inequalities in mortality: New Zealand (1981–99). J Epidemiol Community Health.

[14] Chang Y, Kim I, Bahk J, Khang YH (2018). Trends in inequality in cigarette smoking prevalence by income according to recent anti-smoking policies in Korea: use of three National Surveys. J Prev Med Public Health.

[15] Cho HJ, Song YM, Smith GD, Ebrahim S (2004). Trends in socio-economic differentials in cigarette smoking behaviour between 1990 and 1998: a large prospective study in Korean men. Public Health.

[16] Jang TW, Kim HR, Choi SE, Yim HW, Lee HE, Myong JP (2012). Smoking rate trends in Korean occupational groups: analysis of KNHANES 1998-2009 data. J Occup Health.

[17] Khang Y-H, Cho H-J (2006). Socioeconomic inequality in cigarette smoking: trends by gender, age, and socioeconomic position in South Korea, 1989–2003. Prev Med.

[18] Khang Y-H, Yun S-C, Cho H-J, Jung-Choi K (2009). The impact of governmental antismoking policy on socioeconomic disparities in cigarette smoking in South Korea. Nicotine Tob Res.

[19] Kim I, Bahk J, Yoon TH, Yun SC, Khang YH (2017). Income differences in smoking Prevalences in 245 districts of South Korea: patterns by area deprivation and urbanity, 2008-2014. J Prev Med Public Health.

[20] Kim I, Bahk J, Kim YY, Lee J, Kang HY, Lee J (2018). Comparison of district-level smoking prevalence and their income gaps from two National Databases: the National Health Screening Database and the community health survey in Korea, 2009-2014. J Korean Med Sci.

[21] Kim YJ, Lee JS, Park J, Choi DS, Kim DM, Lee KH (2017). Trends in socioeconomic inequalities in five major risk factors for cardiovascular disease in the Korean population: a cross-sectional study using data from the Korea National Health and nutrition examination survey, 2001-2014. BMJ Open.

[22] Khang Y-H, Lynch J, Jung-Choi K, Cho H-J (2008). Explaining age specific inequalities in mortality from all causes, cardiovascular disease and Ischaemic heart disease among south Korean male public servants: relative and absolute perspectives. Heart.

[23] The Social Survey of Statistics Korea. Statistics Korea, Daejeon, Korea. 2018. http://mdis.kostat.go.kr/index.do. Accessed 01 Aug 2019.

[24] Spiegelman D, Hertzmark E (2005). Easy SAS calculations for risk or prevalence ratios and differences. Am J Epidemiol.

[25] Harper S, Lynch J. Health inequalities: measurement and decomposition. In: Oakes JM, Kaufman JS, eds. Methods in social epidemiology Second Edition. San Francisco, CA: Jossey-Bass and Pfeiffer Imprint, Wiley; 2017, 91–131.

[26] Mackenbach JP, Kunst AE (1997). Measuring the magnitude of socio-economic inequalities in health: an overview of available measures illustrated with two examples from Europe. Soc Sci Med.

[27] Levy DT, Cho SI, Kim YM, Park S, Suh MK, Kam S (2010). SimSmoke model evaluation of the effect of tobacco control policies in Korea: the unknown success story. Am J Public Health.

[28] Seo MK, Shin YJ, Jung YH, Oh YM, Koh SJ (2003). Evaluation on the effect of National Anti-Smoking Policies in Korea.

[29] Kwak J, Jeong H, Chun S, Bahk JH, Park M, Byun Y (2017). Effectiveness of government anti-smoking policy on non-smoking youth in Korea: a 4-year trend analysis of national survey data. BMJ Open.

[30] Honjo K, Kawachi I (2000). Effects of market liberalisation on smoking in Japan. Tob Control.

[31] Cho HJ, Khang YH, Jun HJ, Kawachi I (2008). Marital status and smoking in Korea: the influence of gender and age. Soc Sci Med.

[32] Rani M, Bonu S, Jha P Nguyen SN, Jamjoum L (2003). Tobacco use in India: prevalence and predictors of smoking and chewing in a national cross sectional household survey. Tob Control.

[33] Yang G, Fan L, Tan J, Qi G, Zhang Y, Samet JH (1999). Smoking in China. Findings of the 1996 National Prevalence Survey. JAMA.

[34] Narayan KMV, Chaadha SL, Hanson RL, Tandon R, Shekhawat S, Fernandes RJ (1996). Prevalence and patterns of smoking in Delhi: cross sectional study. BMJ.

[35] Jenkins CNH, Dai PX, Ngoc DH, Kinh HV, Hoang TT, Bales S (1997). Tobacco use in Vietnam. Prevalence, predictors, and the role of the transnational tobacco corporations. JAMA.

[36] Graham H (1996). Smoking prevalence among women in the European community 1950–1990. Soc Sci Med.

[37] Jung-Choi K-H, Khang Y-H, Cho H-J (2012). Hidden female smokers in Asia: a comparison of self-reported with cotinine-verified smoking prevalence rates in representative national data from an Asian population. Tob Control.

[38] Khang YH, Kim HR. Socioeconomic inequality in mortality using 12-year follow-up data from nationally representative surveys in South Korea. Int J Equity Health. 2016;15:51. 10.1186/s12939-016-0341-9 PMC4802872 27001045

