# Supplementary material for: Long-term trends in smoking prevalence and its socioeconomic inequalities in Korea, 1992–2016
Source: Int J Equity Health. 2019 Sep 18;18:148. doi: 10.1186/s12939-019-1051-x (PMC6751588; doi:10.1186/s12939-019-1051-x)
Supplement: Supplementary file 1 — Additional file 1: Table S1. Numbers and proportions (standard error) of subjects and smokers according to sex, age group, education, occupation, and income tertile: Results from the Social Survey of Statistics Korea. Table S2. Trends in age-standardized smoking prevalence (%) and its 95% confidence intervals (CI) according to sex, age group, education, occupation, and income tertile: Results from the Social Survey of Statistics Korea. (DOCX 51 kb) [file 12939_2019_1051_MOESM1_ESM.docx]

Supplementary Table 1. Numbers and proportions (standard error) of subjects and smokers according to sex, age group, education, occupation, and income tertile: Results from the Social Survey of Statistics Korea

|  | 1992 | | 1995 | | 1999 | | 2003 | | 2006 | | 2008 | | 2010 | | 2012 | | 2014 | | 2016 | |
| --- | --- | --- | --- | --- | --- | --- | --- | --- | --- | --- | --- | --- | --- | --- | --- | --- | --- | --- | --- | --- |
|  | Subjects | Smokers | Subjects | Smokers | Subjects | Smokers | Subjects | Smokers | Subjects | Smokers | Subjects | Smokers | Subjects | Smokers | Subjects | Smokers | Subjects | Smokers | Subjects | Smokers |
| Men (n) | 35,774 | 26,064 | 35,410 | 25,765 | 29,479 | 19,870 | 30,547 | 17,021 | 31,774 | 15,148 | 18,707 | 9,334 | 16,123 | 7,489 | 15,671 | 6,834 | 15,912 | 6,669 | 16,699 | 6,330 |
| Age |  |  |  |  |  |  |  |  |  |  |  |  |  |  |  |  |  |  |  |  |
| 19-34 | 42.6 (0.3) | 44.1 (0.3) | 40.6 (0.3) | 42.5 (0.3) | 35 (0.3) | 37.1 (0.4) | 35.0 (0.3) | 37.8 (0.4) | 32.0 (0.3) | 34.4 (0.5) | 30.0 (0.4) | 31.8 (0.6) | 29.0 (0.4) | 30.4 (0.7) | 28.5 (0.5) | 30.5 (0.7) | 27.9 (0.5) | 27.4 (0.7) | 27.1 (0.5) | 26.1 (0.7) |
| 35-49 | 31.0 (0.3) | 31.2 (0.3) | 33.3 (0.3) | 34.0 (0.3) | 36.1 (0.3) | 38.5 (0.4) | 36.1 (0.3) | 38.8 (0.4) | 36.1 (0.3) | 40.5 (0.4) | 36.2 (0.4) | 40.6 (0.6) | 35.1 (0.4) | 40.5 (0.6) | 33.2 (0.4) | 38.9 (0.7) | 31.9 (0.4) | 39 (0.7) | 31.2 (0.4) | 39.8 (0.8) |
| 50-64 | 19.5 (0.2) | 18.7 (0.3) | 18.9 (0.2) | 17.6 (0.3) | 19.4 (0.2) | 18.2 (0.3) | 19.4 (0.2) | 16.9 (0.3) | 21.1 (0.3) | 18.4 (0.3) | 22.4 (0.3) | 20.2 (0.4) | 24.0 (0.4) | 22.0 (0.5) | 25.4 (0.4) | 23.8 (0.6) | 26.5 (0.4) | 26.5 (0.6) | 27.4 (0.4) | 27.6 (0.7) |
| 65≤ | 6.8 (0.1) | 6.1 (0.2) | 7.3 (0.1) | 5.9 (0.2) | 9.4 (0.2) | 6.2 (0.2) | 9.4 (0.2) | 6.4 (0.2) | 10.8 (0.2) | 6.6 (0.2) | 11.4 (0.2) | 7.4 (0.3) | 11.9 (0.3) | 7.1 (0.3) | 12.9 (0.3) | 6.8 (0.3) | 13.7 (0.3) | 7.1 (0.3) | 14.4 (0.3) | 6.5 (0.3) |
| Education |  |  |  |  |  |  |  |  |  |  |  |  |  |  |  |  |  |  |  |  |
| High school or less | 74.0 (0.3) | 76.4 (0.3) | 70.5 (0.3) | 73.2 (0.3) | 59.1 (0.3) | 69.3 (0.4) | 59.1 (0.3) | 62.8 (0.4) | 59.0 (0.3) | 60.1 (0.5) | 52.5 (0.4) | 56.0 (0.6) | 50.2 (0.5) | 53.9 (0.7) | 49.8 (0.5) | 53.0 (0.7) | 47.9 (0.5) | 52.8 (0.7) | 46.6 (0.5) | 50.6 (0.8) |
| College or higher | 26.0 (0.3) | 23.6 (0.3) | 29.5 (0.3) | 26.8 (0.3) | 40.9 (0.3) | 30.7 (0.4) | 40.9 (0.3) | 37.2 (0.4) | 41.0 (0.3) | 39.9 (0.5) | 47.5 (0.4) | 44.0 (0.6) | 49.8 (0.5) | 46.1 (0.7) | 50.2 (0.5) | 47.0 (0.7) | 52.1 (0.5) | 47.2 (0.7) | 53.4 (0.5) | 49.4 (0.8) |
| Occupation |  |  |  |  |  |  |  |  |  |  |  |  |  |  |  |  |  |  |  |  |
| Non-manual | 20.4 (0.2) | 19.0 (0.3) | 17.0 (0.2) | 15.5 (0.3) | 27.3 (0.3) | 23.4 (0.4) | 27.3 (0.3) | 25.8 (0.4) | 25.5 (0.3) | 25.5 (0.4) | 30.8 (0.4) | 29.0 (0.5) | 28.2 (0.4) | 26.4 (0.6) | 27.2 (0.4) | 25.5 (0.6) | 28.0 (0.4) | 25.3 (0.7) | 27.3 (0.4) | 25.9 (0.7) |
| Manual | 61.9 (0.3) | 66.1 (0.3) | 65.4 (0.3) | 69.0 (0.3) | 50.0 (0.3) | 57.6 (0.4) | 50.0 (0.3) | 54.8 (0.4) | 46.4 (0.3) | 55.2 (0.5) | 45.7 (0.4) | 51.8 (0.6) | 44.9 (0.5) | 51.5 (0.7) | 46.2 (0.5) | 53.7 (0.7) | 45.4 (0.5) | 53.6 (0.7) | 45.1 (0.5) | 52.6 (0.8) |
| Others | 17.7 (0.2) | 14.9 (0.2) | 17.7 (0.2) | 15.5 (0.2) | 22.7 (0.3) | 18.9 (0.3) | 22.7 (0.3) | 19.3 (0.3) | 28.1 (0.3) | 19.2 (0.4) | 23.5 (0.4) | 19.2 (0.5) | 26.9 (0.4) | 22.1 (0.6) | 26.6 (0.4) | 20.8 (0.6) | 26.5 (0.4) | 21.1 (0.6) | 27.6 (0.4) | 21.5 (0.6) |
| Income |  |  |  |  |  |  |  |  |  |  |  |  |  |  |  |  |  |  |  |  |
| Ⅰ (lowest) |  |  |  |  |  | 34.0 (0.4) |  |  | 31.0 (0.3) | 33.7 (0.4) | 31.6 (0.4) | 32.9 (0.5) | 30.2 (0.4) | 32.4 (0.6) | 33.9 (0.4) | 36.3 (0.7) | 32.2 (0.4) | 34.6 (0.7) | 30.7 (0.4) | 33.6 (0.7) |
| Ⅱ |  |  |  |  |  | 31.5 (0.4) |  |  | 34.9 (0.3) | 35.0 (0.4) | 33.5 (0.4) | 35.1 (0.6) | 34.2 (0.4) | 36.1 (0.6) | 31.2 (0.4) | 32.4 (0.7) | 33.8 (0.4) | 34.5 (0.7) | 36.0 (0.5) | 35.6 (0.7) |
| Ⅲ (highest) |  |  |  |  |  | 34.5 (0.4) |  |  | 34.2 (0.3) | 31.4 (0.4) | 34.8 (0.4) | 32.0 (0.6) | 35.6 (0.4) | 31.5 (0.6) | 34.9 (0.4) | 31.3 (0.7) | 34.1 (0.5) | 30.9 (0.7) | 33.3 (0.5) | 30.8 (0.7) |
|  |  |  |  |  |  |  |  |  |  |  |  |  |  |  |  |  |  |  |  |  |
| Women (n) | 40,767 | 2,440 | 40,097 | 2,415 | 33,375 | 1,538 | 35,039 | 1,313 | 36,112 | 1,275 | 20,851 | 627 | 18,011 | 551 | 17,678 | 646 | 17,981 | 543 | 18,859 | 514 |
| Age |  |  |  |  |  |  |  |  |  |  |  |  |  |  |  |  |  |  |  |  |
| 19-34 | 42.4 (0.3) | 11.7 (0.7) | 40.5 (0.3) | 16.1 (0.8) | 34.8 (0.3) | 21.0 (1.2) | 34.8 (0.3) | 26.3 (1.4) | 31.7 (0.3) | 24.8 (1.5) | 29.7 (0.4) | 34.3 (2.3) | 28 (0.4) | 34.4 (2.5) | 27.2 (0.4) | 30.7 (2.2) | 26.4 (0.4) | 31.9 (2.4) | 25.3 (0.4) | 32.9 (2.7) |
| 35-49 | 27.7 (0.2) | 13.8 (0.8) | 29.5 (0.3) | 16.3 (0.8) | 32.9 (0.3) | 20.7 (1.2) | 32.9 (0.3) | 25.2 (1.3) | 33.2 (0.3) | 29.7 (1.4) | 33.1 (0.4) | 27.7 (2.0) | 32.4 (0.4) | 26.0 (2.1) | 30.8 (0.4) | 29.5 (2.1) | 29.7 (0.4) | 25.5 (2.2) | 29.0 (0.4) | 28.8 (2.5) |
| 50-64 | 19.3 (0.2) | 31.4 (1.0) | 18.8 (0.2) | 25.9 (1.0) | 18.8 (0.2) | 20.0 (1.1) | 18.8 (0.2) | 16.6 (1.1) | 20.2 (0.2) | 18.7 (1.2) | 21.4 (0.3) | 18.7 (1.7) | 23.2 (0.4) | 19.4 (1.9) | 24.6 (0.4) | 26.2 (2.0) | 25.6 (0.4) | 29.4 (2.3) | 26.6 (0.4) | 26.7 (2.3) |
| 65≤ | 10.6 (0.2) | 43.1 (1.1) | 11.2 (0.2) | 41.8 (1.1) | 13.5 (0.2) | 38.2 (1.4) | 13.5 (0.2) | 31.9 (1.4) | 14.9 (0.2) | 26.8 (1.3) | 15.8 (0.3) | 19.3 (1.6) | 16.5 (0.3) | 20.1 (1.8) | 17.4 (0.3) | 13.6 (1.4) | 18.2 (0.3) | 13.2 (1.5) | 19.1 (0.3) | 11.5 (1.5) |
| Education |  |  |  |  |  |  |  |  |  |  |  |  |  |  |  |  |  |  |  |  |
| High school or less | 86.3 (0.2) | 97.4 (0.4) | 83.5 (0.2) | 95.9 (0.5) | 72.1 (0.3) | 91.4 (0.9) | 72.1 (0.3) | 89.3 (1.0) | 70 (0.3) | 85.4 (1.2) | 65.0 (0.4) | 78.3 (2.0) | 63.4 (0.4) | 76.4 (2.2) | 60.9 (0.4) | 74.4 (2.0) | 58.9 (0.4) | 72.3 (2.3) | 57.4 (0.5) | 68.9 (2.6) |
| College or higher | 13.7 (0.2) | 2.6 (0.4) | 16.5 (0.2) | 4.1 (0.5) | 27.9 (0.3) | 8.6 (0.9) | 27.9 (0.3) | 10.7 (1.0) | 30 (0.3) | 14.6 (1.2) | 35.0 (0.4) | 21.7 (2.0) | 36.6 (0.4) | 23.6 (2.2) | 39.1 (0.4) | 25.6 (2.0) | 41.1 (0.4) | 27.7 (2.3) | 42.6 (0.5) | 31.1 (2.6) |
| Occupation |  |  |  |  |  |  |  |  |  |  |  |  |  |  |  |  |  |  |  |  |
| Non-manual | 11.4 (0.2) | 3.0 (0.4) | 6.0 (0.1) | 1.3 (0.3) | 16.9 (0.2) | 6.2 (0.8) | 16.9 (0.2) | 6.6 (0.8) | 18.1 (0.2) | 11.4 (1.1) | 18.6 (0.3) | 12.7 (1.7) | 18.9 (0.4) | 14.2 (1.9) | 18.8 (0.4) | 12.8 (1.6) | 20 (0.4) | 11.9 (1.8) | 21.1 (0.4) | 15.4 (2.1) |
| Manual | 39.4 (0.3) | 41.4 (1.1) | 46.4 (0.3) | 44.5 (1.1) | 34.3 (0.3) | 42.7 (1.4) | 34.3 (0.3) | 39.3 (1.5) | 31.6 (0.3) | 39.6 (1.5) | 25.6 (0.3) | 42.2 (2.2) | 27.1 (0.4) | 35.7 (2.4) | 28.8 (0.4) | 44.9 (2.3) | 28.3 (0.4) | 43.6 (2.5) | 28.2 (0.4) | 44.6 (2.7) |
| Others | 49.2 (0.3) | 55.6 (1.1) | 47.6 (0.3) | 54.2 (1.1) | 48.8 (0.3) | 51.1 (1.4) | 48.8 (0.3) | 54.1 (1.5) | 50.4 (0.3) | 48.9 (1.6) | 55.8 (0.4) | 45.0 (2.2) | 54.1 (0.4) | 50.1 (2.5) | 52.4 (0.4) | 42.2 (2.3) | 51.6 (0.4) | 44.6 (2.5) | 50.7 (0.5) | 40.0 (2.6) |
| Income |  |  |  |  |  |  |  |  |  |  |  |  |  |  |  |  |  |  |  |  |
| Ⅰ (lowest) |  |  |  |  |  | 36.8 (1.4) |  |  | 31.6 (0.3) | 42.5 (1.5) | 30.4 (0.4) | 40.6 (2.2) | 28.2 (0.4) | 37.2 (2.4) | 33.4 (0.4) | 45.7 (2.3) | 30.4 (0.4) | 41.7 (2.5) | 29.4 (0.4) | 38.4 (2.6) |
| Ⅱ |  |  |  |  |  | 32.0 (1.3) |  |  | 32.8 (0.3) | 30.7 (1.5) | 33.1 (0.4) | 32.3 (2.1) | 36.2 (0.4) | 35.5 (2.3) | 32.6 (0.4) | 34.0 (2.2) | 35.5 (0.4) | 35.1 (2.4) | 35.2 (0.4) | 35.8 (2.6) |
| Ⅲ (highest) |  |  |  |  |  | 31.2 (1.4) |  |  | 35.6 (0.3) | 26.8 (1.4) | 36.6 (0.4) | 27.1 (2.1) | 35.6 (0.4) | 27.3 (2.3) | 34.0 (0.4) | 20.3 (1.9) | 34.1 (0.4) | 23.2 (2.2) | 35.4 (0.4) | 25.8 (2.4) |

Supplementary Table 2. Trends in age-standardized smoking prevalence (%) and its 95% confidence intervals (CI) according to sex, age group, education, occupation, and income tertile: Results from the Social Survey of Statistics Korea

|  | 1992 | | 1995 | | 1999 | | 2003 | | 2006 | | 2008 | | 2010 | | 2012 | | 2014 | | 2016 | |
| --- | --- | --- | --- | --- | --- | --- | --- | --- | --- | --- | --- | --- | --- | --- | --- | --- | --- | --- | --- | --- |
|  | Smoking Prevalence | 95% CI | Smoking Prevalence | 95% CI | Smoking Prevalence | 95% CI | Smoking Prevalence | 95% CI | Smoking Prevalence | 95% CI | Smoking Prevalence | 95% CI | Smoking Prevalence | 95% CI | Smoking Prevalence | 95% CI | Smoking Prevalence | 95% CI | Smoking Prevalence | 95% CI |
| Men |  |  |  |  |  |  |  |  |  |  |  |  |  |  |  |  |  |  |  |  |
| Total | 71.7 | (71.1-72.2) | 71.5 | (70.9-72.0) | 66.3 | (65.7-66.9) | 55.0 | (54.4-55.6) | 51.2 | (50.6-51.8) | 50.2 | (49.4-51.0) | 47.0 | (46.0-47.9) | 44.7 | (43.8-45.7) | 43.2 | (42.3-44.2) | 39.7 | (38.7-40.6) |
| Age |  |  |  |  |  |  |  |  |  |  |  |  |  |  |  |  |  |  |  |  |
| 19-34 | 74.6 | (73.8-75.3) | 75.7 | (74.9-76.5) | 70.8 | (69.7-71.8) | 59.5 | (58.4-60.6) | 54.1 | (50.1-53.4) | 51.8 | (46.1-49.9) | 48.0 | (46.1-49.9) | 46.8 | (44.9-48.7) | 41.6 | (39.7-43.5) | 37.7 | (35.8-39.6) |
| 35-49 | 72.6 | (71.6-73.5) | 73.9 | (73.0-74.8) | 71.4 | (70.4-72.4) | 60.1 | (59.1-61.1) | 58.1 | (57.1-59.1) | 56.5 | (55.2-57.8) | 54.3 | (52.8-55.8) | 52.3 | (50.8-53.8) | 52.8 | (51.1-54.4) | 49.9 | (48.2-51.6) |
| 50-64 | 69.2 | (68.0-70.4) | 67.7 | (66.5-68.9) | 60.0 | (58.6-61.4) | 48.7 | (47.3-50.0) | 45.2 | (43.9-46.5) | 45.3 | (43.7-46.9) | 42.7 | (41.0-44.5) | 41.3 | (39.6-43.0) | 42.7 | (41.0-44.3) | 39.6 | (37.9-41.3) |
| ≥65 | 64.0 | (61.9-66.1) | 58.5 | (56.5-60.5) | 48.7 | (46.5-50.9) | 37.7 | (35.9-39.5) | 31.2 | (29.6-32.9) | 32.2 | (30.2-34.2) | 28.2 | (26.2-30.2) | 23.5 | (21.6-25.3) | 22.4 | (20.8-24.1) | 17.9 | (16.3-19.5) |
| Education |  |  |  |  |  |  |  |  |  |  |  |  |  |  |  |  |  |  |  |  |
| High school or less | 74.4 | (73.8-75.0) | 74.9 | (74.3-75.5) | 71.0 | (70.3-71.7) | 61.7 | (50.9-62.5) | 58.7 | (57.8-59.7) | 58.0 | (56.7-59.3) | 56.2 | (54.6-57.7) | 52.9 | (51.3-54.5) | 54.0 | (52.2-55.7) | 49.7 | (47.9-51.4) |
| College or higher | 62.0 | (60.3-63.8) | 60.1 | (59.5-62.5) | 56.6 | (55.2-58.0) | 46.6 | (45.4-47.7) | 44.2 | (43.0-45.3) | 43.5 | (42.2-44.8) | 40.9 | (39.6-42.2) | 38.6 | (37.3-39.9) | 36.9 | (35.6-38.2) | 34.3 | (33.1-35.6) |
| Occupation |  |  |  |  |  |  |  |  |  |  |  |  |  |  |  |  |  |  |  |  |
| Non-manual | 63.0 | (61.1-65.0) | 63.0 | (61.1-65.0) | 61.0 | (59.0-63.1) | 49.1 | (47.3-51.0) | 44.4 | (42.7-46.1) | 44.7 | (42.8-46.7) | 41.1 | (39.0-43.2) | 38.4 | (36.2-40.6) | 36.6 | (34.3-38.8) | 35.0 | (32.9-37.2) |
| Manual | 76.9 | (76.2-77.6) | 75.5 | (74.7-76.1) | 72.1 | (71.2-72.9) | 60.9 | (60.1-61.8) | 57.0 | (56.1-57.9) | 57.2 | (56.0-58.5) | 54.2 | (52.8-55.6) | 52.0 | (50.6-53.4) | 50.6 | (49.1-52.0) | 45.8 | (44.3-47.2) |
| Others | 65.4 | (63.5-67.3) | 68.8 | (67.1-70.6) | 64.3 | (62.8-65.8) | 55.0 | (53.4-56.6) | 52.3 | (50.7-53.9) | 49.3 | (47.2-51.5) | 45.7 | (43.6-47.8) | 42.5 | (40.3-44.6) | 43.7 | (41.4-45.9) | 38.7 | (36.4-41.0) |
| Income |  |  |  |  |  |  |  |  |  |  |  |  |  |  |  |  |  |  |  |  |
| I (lowest) |  |  |  |  |  | 68.8 | (67.7-69.8) |  | 55.7 | (54.6-56.8) | 53.2 | (51.8-54.7) | 51.2 | (49.6-52.8) | 48.4 | (46.9-50.0) | 46.6 | (45.0-48.2) | 43.2 | (41.6-44.9) |
| II |  |  |  |  |  | 66.9 | (65.8-67.9) |  | 51.4 | (50.3-52.5) | 51.8 | (50.3-53.2) | 48.5 | (47.0-50.1) | 46.0 | (44.3-47.7) | 44.5 | (42.9-46.1) | 39.6 | (38.0-41.2) |
| III (highest) |  |  |  |  |  | 63.6 | (62.5-64.6) |  | 47.0 | (45.8-48.1) | 46.2 | (44.8-47.6) | 42.1 | (40.6-43.7) | 40.2 | (38.6-41.7) | 39.0 | (37.4-40.6) | 36.5 | (34.8-38.1) |
|  | | | | | | | | | | | | | | | | | | | | |
| Women |  |  |  |  |  |  |  |  |  |  |  |  |  |  |  |  |  |  |  |  |
| Total | 6.5 | (6.3-6.8) | 6.3 | (6.0-6.6) | 4.6 | (4.3-4.8) | 3.8 | (3.6-4.0) | 3.8 | (3.5-4.0) | 3.1 | (2.9-3.4) | 3.1 | (2.8-3.4) | 4.0 | (3.6-4.3) | 3.4 | (3.1-3.8) | 3.3 | (2.9-3.6) |
| Age |  |  |  |  |  |  |  |  |  |  |  |  |  |  |  |  |  |  |  |  |
| 19-34 | 1.6 | (1.4-1.9) | 2.3 | (2.1-2.6) | 2.1 | (1.8-2.4) | 2.9 | (2.5-3.2) | 3.0 | (2.6-3.4) | 3.6 | (3.0-4.2) | 3.9 | (3.2-4.6) | 4.5 | (3.7-5.2) | 4.1 | (3.3-4.8) | 4.0 | (3.2-4.8) |
| 35-49 | 3.0 | (2.6-3.3) | 3.3 | (3.0-3.7) | 2.9 | (2.5-3.4) | 2.9 | (2.6-3.2) | 3.4 | (3.1-3.8) | 2.6 | (2.2-3.0) | 2.5 | (2.0-2.9) | 3.8 | (3.2-4.4) | 2.9 | (2.3-3.4) | 3.1 | (2.5-3.7) |
| 50-64 | 9.6 | (8.9-10.3) | 7.9 | (7.2-8.5) | 5.0 | (4.3-5.7) | 3.3 | (2.9-3.8) | 3.6 | (3.1-4.1) | 2.8 | (2.2-3.3) | 2.6 | (2.0-3.1) | 4.1 | (3.4-4.8) | 3.8 | (3.1-4.5) | 3.1 | (2.5-3.7) |
| ≥65 | 24.1 | (22.7-25.4) | 22.0 | (20.7-23.3) | 13.1 | (11.9-14.3) | 9.0 | (8.1-9.8) | 6.8 | (6.1-7.6) | 3.8 | (3.1-4.5) | 3.6 | (2.9-4.3) | 3.0 | (2.4-3.6) | 2.3 | (1.8-2.8) | 1.9 | (1.4-2.5) |
| Education |  |  |  |  |  |  |  |  |  |  |  |  |  |  |  |  |  |  |  |  |
| High school or less | 6.7 | (6.4-7.0) | 6.5 | (6.3-6.8) | 5.0 | (4.7-5.2) | 4.7 | (4.4-5.0) | 5.0 | (4.6-5.4) | 4.6 | (4.0-5.2) | 5.0 | (4.2-5.8) | 6.4 | (5.5-7.3) | 5.5 | (4.7-6.4) | 5.5 | (4.5-6.5) |
| College or higher | 3.0 | (1.3-4.6) | 1.5 | (1.0-2.1) | 1.8 | (1.2-2.5) | 1.5 | (1.1-2.0) | 1.5 | (1.2-1.8) | 1.5 | (1.1-1.8) | 2.0 | (1.3-2.8) | 2.4 | (1.8-2.9) | 1.9 | (1.5-2.2) | 2.1 | (1.4-2.8) |
| Occupation |  |  |  |  |  |  |  |  |  |  |  |  |  |  |  |  |  |  |  |  |
| Non-manual | 6.4 | (4.3-8.5) | 2.0 | (0.7-3.4) | 3.8 | (0.6-6.9) | 1.9 | (1.0-2.8) | 1.8 | (1.3-2.2) | 1.7 | (1.2-2.2) | 2.0 | (1.4-2.7) | 2.7 | (1.7-3.7) | 1.6 | (1.1-2.2) | 1.9 | (1.3-2.4) |
| Manual | 7.3 | (6.8-7.8) | 6.9 | (6.4-7.3) | 6.0 | (5.5-6.5) | 5.3 | (4.7-5.8) | 5.1 | (4.5-5.6) | 6.4 | (5.4-7.4) | 4.8 | (3.9-5.7) | 7.2 | (6.1-8.3) | 6.3 | (5.2-7.3) | 5.8 | (4.7-6.9) |
| Others | 6.0 | (5.7-6.4) | 5.8 | (5.5-6.2) | 4.0 | (3.7-4.4) | 3.7 | (3.4-4.1) | 3.5 | (3.1-3.8) | 2.4 | (2.1-2.7) | 2.8 | (2.4-3.2) | 3.2 | (2.7-3.6) | 2.9 | (2.4-3.3) | 2.6 | (2.1-3.1) |
| Income |  |  |  |  |  |  |  |  |  |  |  |  |  |  |  |  |  |  |  |  |
| I (lowest) |  |  |  |  |  | 5.1 | (4.7-5.5) |  | 5.1 | (4.6-5.5) | 4.3 | (3.7-4.8) | 4.1 | (3.5-4.8) | 5.3 | (4.6-6.0) | 4.6 | (3.9-5.3) | 4.1 | (3.4-4.8) |
| II |  |  |  |  |  | 4.7 | (4.2-5.1) |  | 3.6 | (3.2-4.0) | 3.1 | (2.6-3.6) | 2.9 | (2.4-3.4) | 4.1 | (3.5-4.8) | 3.3 | (2.8-3.9) | 3.3 | (2.7-3.9) |
| III (highest) |  |  |  |  |  | 4.1 | (3.7-4.5) |  | 2.7 | (2.4-3.1) | 2.3 | (1.9-2.7) | 2.4 | (1.9-2.9) | 2.4 | (1.9-2.9) | 2.3 | (1.8-2.8) | 2.4 | (1.9-2.9) |
|  | | | | | | | | | | | | | | | | | | | | |
